# Three tests of the Vulnerability-Stress-Adaptation Model: Independent prediction, mediation, and generalizability

**DOI:** 10.3389/fpsyg.2022.921485

**Published:** 2022-07-28

**Authors:** Jaclyn M. Ross, Teresa P. Nguyen, Benjamin R. Karney, Thomas N. Bradbury

**Affiliations:** ^1^Department of Psychiatry, University of Illinois at Chicago, Chicago, IL, United States; ^2^Department of Psychology, Sonoma State University, Rohnert Park, CA, United States; ^3^Department of Psychology, University of California, Los Angeles, Los Angeles, CA, United States

**Keywords:** newlywed, longitudinal, communication, stress, marriage

## Abstract

**Objective:**

Efforts to understand why some marriages thrive while others falter are (a) not well integrated conceptually and (b) rely heavily on data collected from White middle-class samples. The Vulnerability-Stress-Adaptation Model (VSA; Karney and Bradbury, 1995) is used here to integrate prior efforts and is tested using data collected from couples living with low incomes.

**Background:**

The VSA Model assumes (a) that enduring vulnerabilities, stress, and couple communication account for unique variance in relationship satisfaction, (b) that communication mediates the effects of vulnerabilities and stress on satisfaction, and (c) that the predictors of satisfaction generalize across socioeconomic levels. To date, these assumptions remain untested.

**Materials and methods:**

With 388 couples from diverse backgrounds (88% Black or Hispanic), we used latent variable structural equation models to examine enduring vulnerabilities, chronic stress, and observed communication as predictors of 4-wave, 27-month satisfaction trajectories, first as main effects and then interacting with a validated 10-item index of sociodemographic risk.

**Results:**

(a) The three variable sets independently predict satisfaction trajectories; (b) couple communication does not mediate the effects of enduring vulnerabilities or stress on satisfaction; and (c) in 19% of tests, effects were stronger among couples with higher sociodemographic risk.

**Conclusion:**

Effects of established predictor domains on satisfaction replicate in a diverse sample of newlywed couples, and most findings generalize across levels of sociodemographic risk. The failure of couple communication to mediate effects of enduring personal vulnerabilities and stress raises new questions about how these two domains undermine committed partnerships.

## Introduction

Because people commit to, and abandon, intimate partnerships with great regularity, hundreds of studies aim to explain how social bonds that were once fulfilling become sources of frustration and regret. Thoughtful application of observational methods characterizes much of this work, thereby illuminating how a wide range of complex interpersonal processes — including negative reinforcement and coercion (e.g., Jacobson and Margolin, 1979), lack of accommodation in response to relationship-threatening actions (e.g., Rusbult et al., 1991), insensitive responses to disclosures (e.g., Cutrona, 1996), and dysregulated emotion (e.g., Gottman and Levenson, 1986) — contribute to later relationship distress. Though unusually generative, at least two major blindspots can be identified in this literature. First, in the vast majority of studies, samples are comprised of couples who are white, well-educated, relatively affluent, and financially stable (Williamson et al., 2021), thus limiting the population to whom findings can be generalized and potentially constraining the effects of preventive and therapeutic interventions that are based on this work (e.g., Heyman et al., 2019). Second, heavy emphasis on interpersonal processes has overshadowed consideration of plausible alternative explanations for how relationships change. Some argue, for example, that interpersonal processes are simply a manifestation of potent up-stream individual differences (e.g., personality traits, early experiences with caregivers, parental divorce) which are themselves the root causes of relationship dysfunction (e.g., Bowlby, 1979; Kelly and Conley, 1987; Amato, 1996). Others look outside the relationship, proposing that normative life events (e.g., the transition to parenthood) and chronic and acute stress (e.g., discrimination, illness, work demands, financial concerns, parenting) undermine relationship maintenance and increase risk for distress and dissolution (e.g., Conger et al., 1990; Bodenmann, 2005). The goal of this work is to address these two blindspots directly, by examining the independent effects of interpersonal processes, individual difference factors, and stress on the relationship satisfaction trajectories experienced by a diverse set of couples recruited from communities characterized by lower incomes.

With the Vulnerability-Stress-Adaptation (VSA) Model, Karney and Bradbury (1995) sought to integrate these three broad domains of influence, hypothesizing that relationship outcomes are governed by (a) *adaptive processes*, or dyad-level patterns of behavioral interdependence, (b) partners’ *enduring vulnerabilities* (i.e., the early experiences and stable traits that a person would bring to any relationship), and (c) *stress* (i.e., the chronic circumstances and acute events that demand partners’ regulatory efforts). The VSA model further asserts that enduring personal vulnerabilities and stress will exert only indirect effects on relationship outcomes, as their influence is expected to be *routed through* the behavioral interdependencies and coordinated actions of the two partners. The VSA model therefore assigns interpersonal processes a central role in determining the course of couple relationships, while also assuming that couples will vary in their adaptive processes because of partners’ vulnerabilities and the stressful circumstances to which couples are exposed. According to this view, marriages become less rewarding over time not simply because of the adverse traits and experiences that partners bring to the relationship, and not simply because of the stresses and strains that partners encounter once in the relationship, but because vulnerabilities and stress outstrip partners’ capacity to create an adaptable and appropriately responsive behavioral repertoire that is necessary to support positive appraisals of their relationship.

Extensive research supports basic bivariate paths within the VSA framework. Consistent with model predictions, for example, meta-analyses and large-scale studies corroborate expected main effects of enduring vulnerabilities (e.g., negative emotionality, Malouff et al., 2010; parental divorce, Dronkers and Härkönen, 2008), stress (Randall and Bodenmann, 2009), and couple communication (Woodin, 2011) on relationship quality and longevity (for recent reviews and analyses, see McNulty et al., 2021; Righetti et al., 2022). Other studies document associations among the three main model components. For example, partners with divorced parents and higher levels of impulsive and neurotic traits display more negative behaviors in couple interactions (e.g., Sanders et al., 1999; Weiss et al., 2018), and partners exposed to higher levels of stress are less effective and more negative when providing social support (Bodenmann et al., 2015) and resolving relationship differences (Williamson et al., 2013). And, at least when considered separately, newlyweds’ enduring vulnerabilities (*viz*., neuroticism, trait anger, and low self-esteem), stress, and adaptive processes displayed during a relationship problem-solving task all predict relationship outcomes 4 and 10 years later (Lavner and Bradbury, 2010). In principle, the VSA model applies to couples regardless of their ethnicity and socioeconomic standing, though in practice the above links are rarely tested with minoritized couples and couples living with low incomes.

Our first aim is to test whether the three main VSA model components make unique and independent contributions to marital outcomes, using a diverse sample of newlywed couples. Although the above-noted bivariate associations appear to support this assumption of independent contributions, plausible alternative models include the possibility that one domain will override the predictive effects of all other domains, either because a domain encompasses developmental phenomena that are broadly influential in individuals’ lives (e.g., traits and vulnerabilities, as proposed by Kelly and Conley, 1987) or because a domain appears to be most causally proximal to judgments of relationship satisfaction (e.g., adaptive processes; as suggested by Jacobson and Margolin, 1979). Examining the relative contributions of VSA model components has important implications for social policies aimed at helping families: e.g., if associations between stress and relationship outcomes are eliminated after controlling for adaptive processes, then a stronger case can be made for couple communication as the primary target of large-scale intervention efforts. In contrast, if stress predicts relationship outcomes independent of adaptive processes, then outside demands on couples’ resources also emerge as a viable target for strengthening committed relationships—particulary if the expected association between adaptive processes and relationship satisfaction falls to nonsignificance.

While enduring vulnerabilities and stress appear to contribute to between-couple variance in relationship processes and outcomes, fewer studies test the critical VSA assumption that relationship processes will mediate these associations. Evidence from smaller studies supports this mediational role of adaptive processes (e.g., Story et al., 2004; Fisher and McNulty, 2008) as does a synthesis of ten longitudinal studies (representing 1104 couples) conducted by McNulty et al. (2021), which showed that higher levels of attachment anxiety predicted slower declines in satisfaction via positive effects of attachment anxiety on observed behavioral engagement. Building from these results, our second aim is to test the assumption that adaptive processes mediate effects of enduring vulnerabilities and stress on relationship outcomes, a possibility tested against the alternative view that partners’ satisfaction reports are responsive to all three predictor domains, with no mediation by adaptive processes. The latter finding would be consistent with the idea that stress directly alters perceptions of relationship quality by elevating expectations of what constitutes a fulfilling relationship or that early experiences with parental divorce or caregiving lead partners to appraise their relationships in fundamentally different ways.

A third source of ambiguity within the VSA model concerns its generalizability across couples with varying social and economic resources. VSA model paths are assumed to be broadly applicable across the economic spectrum (Karney and Bradbury, 1995), yet the model makes no special provisions for couples’ socioedemographic risk status, and the intersection between such risk factors and VSA model predictions remains undeveloped. This may be a significant oversight, insofar as relationship distress and dissolution are known to be more common among couples with fewer years of formal education, lower incomes, greater use of government services, and greater social isolation (Bramlett and Mosher, 2002; Copen et al., 2012; Karney, 2021; Pietromonaco and Overall, 2022). How might the VSA model accommodate couples’ social and economic disparities? One possibility is that, regardless of sociodemographic risk, all couple relationships are governed by enduring vulnerabilities, stress, and adaptive processes in essentially the same way. While couples living with lower incomes, fewer years of formal education, or fewer close social ties might enter their relationships with more enduring vulnerabilities or might encounter more stress, for example, the magnitude of the paths linking vulnerabilities, stress, and adaptive processes to couple outcomes might be uniform across levels of risk. However, such a prediction overlooks a second possibility, that couples with differing levels of sociodeomographic risk may be *qualitatively* distinct from one another in how their relationships develop. Indeed, abuse in childhood, an enduring vulnerability, exerts stronger effects on relationship distress among women with less social capital (Liang et al., 2006), and the transition to parenthood, a normative stressor, exerts stronger effects on satisfaction among couples with lower incomes (Doss et al., 2009). If the effects of model components on satisfaction are moderated by sociodemographic risk, as these studies suggest, then the VSA model could be revised in ways that capture the experiences of a wider spectrum of couples. Our third aim is to distinguish between these two possibilities, by testing the moderating role of sociodemographic risk on paths linking enduring vulnerabilities, chronic stress, and adaptive processes to relationship satisfaction.

## The present study

To test our three main aims, we collected self-report data on three prominent enduring vulnerabilities (abuse history, experiences in the family-of-origin, and symptoms of depression); self-report data on several common forms of chronic stress (e.g., stress arising from close friendships, family relationships, work, finances, and health); and observational data from three 8-minute, in-home couple interaction tasks that were later coded for positivity, negativity, and communication effectiveness. Although observational data on communication behaviors may not provide a fully comprehensive operationalization of adaptive processes, we rely on them here because communication behaviors are a robust correlate of relationship satisfaction (Woodin, 2011), and because observational data reduce shared method variance when compared to self-report assessments of comparable constructs. To assess the potential moderating role of sociodemographic risk, we employed a 10-item index developed by Amato (2014) that defines risk in terms of partners’ age at marriage, levels of formal education, employment status, income, use of public assistance, and access to others during an emergency. To address concerns about the lack of racial and economic diversity in studies of marriage, we used census data to sample couples living in communities with low incomes, drawing from public marriage licenses to recruit first-time newlywed couples; doing so also helps to ensure sufficient variability on the risk index, which is critical for effective testing of our generalizability hypothesis (Aim 3). Finally, 4 self-report assessments of relationship satisfaction, collected at 9-month intervals, were conducted to enable longitudinal analysis of the first 27 months of marriage. Multiple waves of satisfaction data allow us to separate between-person levels of satisfaction from within-person changes in satisfaction, and because most findings in the literature are between-person effects (e.g., Lavner et al., 2014), we expect level (intercept) effects to predominate here as well.

## Materials and methods

### Participants

The 431 newlywed couples (862 spouses) recruited to participate had been married an average of 4.8 months; 38.5% entered marriage with children. Men and women averaged 27.9 (*SD* = 5.8) and 26.3 (*SD* = 5.0) years of age. The mean household income for the sample at baseline in 2009 was $51,290 (*SD* = $27,711), while the median household income was $47,500, with incomes ranging from $2,500 to $100,000+ per year. Note that the median household income for Los Angeles County residents in 2009 was $54,375 (U.S. Census Bureau), suggesting that our sampling procedure successfully included a range of couples with varying socioeconomic statuses. Couples were Black (12%), White (12%), and Hispanic (76%), in proportions roughly comparable to those of people living in poverty in Los Angeles County (12.9%, 14.7%, and 60.5%, respectively; United States Census Bureau, 2002). Missing and incomplete data, in the interaction task and/or the self-report questionnaires, reduced the initial sample of 431 couples by 43 couples, leaving 388 couples (776 spouses) available for analysis, 36.3% of whom entered marriage with children. For comparions of couples entering marriage with and without children, see Lavner et al. (2020).

### Procedure

#### Recruitment

Sampling was undertaken to yield a group of first-married newlywed couples of the same ethnicity, living in low-income neighborhoods. Recently married couples were identified through names and addresses on marriage license applications. Addresses were matched with census data to identify applicants living in low-income communities, defined as census block groups wherein the median household income was no more than 160% of the 1999 federal poverty level for a four-person family. Next, names on the licenses were weighted using data from a Bayesian Census Surname Combination (BCSC), which integrates census and surname information to produce a multinomial probability of membership in each of four categories (Hispanic, Black, Asian, White/Other). Couples were selected from the population of recently married couples using probabilities proportionate to the ratio of target prevalence to the population prevalence, weighted by the couple’s average estimated probability of being Hispanic, Black, or White, which are the three largest groups among people living in poverty in Los Angeles County (United States Census Bureau, 2002). These couples were telephoned and screened to ensure that they had married, that neither partner had been previously married, and that both spouses identified as Hispanic, Black, or White.

#### Assessments

Couples were visited in their homes four times, once every 9 months, by two trained interviewers who described the IRB-approved study and obtained written informed consent from each participant. Interviewers took spouses to separate areas in their home to orally administer self-report measures at baseline (T1), and identical procedures were used 9 months (T2), 18 months (T3), and 27 months (T4) after baseline. Couples who reported that they had divorced or separated did not complete the interview. Following each interview, couples were debriefed and paid $75 for T1, $100 for T2, $125 for T3, and $150 for T4. Data collection occurred between 2009 and 2013.

Following their individual interviews, partners were reunited for three 8-min discussions. Discussions took place in a location of the couples’ choosing that would enable them to talk privately and without interruption. The first two discussions used procedures designed to assess social support behaviors (Pasch and Bradbury, 1998). One randomly chosen spouse was asked to “talk about something you would like to change about yourself” while the partner was instructed to “be involved in the discussion and respond in whatever way you wish.” Spouses were asked to avoid sources of tension within the relationship. After a short break, a second discussion was held that was identical to the first discussion, with the roles reversed. Common topics included losing weight, making a career change, and dealing with stress. For the third interaction, partners were asked to identify a topic of disagreement in their relationship and to then devote 8 min working toward a mutually satisfying resolution of that topic. Common topics included management of money, chores, communication, and spending time together as a couple.

#### Observational coding

Videotapes were scored by 16 trained coders using the Iowa Family Interaction Rating Scales (IFIRS; Melby et al., 1998). Coders—five of whom were native Spanish speakers—coded only in their native language. The IFIRS gives each participant a single score for each behavioral code at the end of the discussion. This score is determined by the coder based on the frequency and intensity with which the participant exhibits the verbal and nonverbal behavior described in the code. The scores range from 1 to 9, with a score of 1 indicating that the behavior did not occur. In general, a score of 3 indicates that “the behavior almost never occurs or occurs just once and is of low intensity,” a score of 5 means “the behavior sometimes occurs and is at a low or moderate level of intensity,” a score of 7 means that “the behavior occurs fairly consistently or is of elevated intensity,” and a score of 9 means “the behavior occurs frequently or with significant intensity” (Melby et al., 1998, pp. 7–8.)

Coders were trained for 10 hours per week for 3 months and were required to pass written and viewing tests with 80% accuracy level before coding, using criterion scores determined by the developers of IFIRS. Coders participated in 2 h of continuing training each week (e.g., coding a tape as a group and watching examples of specific codes) to minimize drift and ensure continued fidelity to IFIRS codes. Coders viewed each interaction three or four times using the Noldus Observer XT coding software, using the built-in capabilities to note behaviors of both spouses. After viewing an interaction, coders used their recorded notations to tabulate the frequency and intensity of each type of behavior and used this information to assign a score for each spouse for each code, using criteria from the IFIRS coding manual (Melby et al., 1998).

To assess reliability, 20% of videos were coded by two randomly chosen coders. Their scores were compared, and any discrepancies greater than one point were resolved by coders working together. Thus the final set of scores used in analyses for the reliability tapes included scores that matched across the two coders during their initial individual coding (when codes were off by 1 point, the score from the randomly designated “primary coder” was used); discrepant scores were replaced by the scores from the second joint coding.

### Measures

#### Enduring vulnerabilities

Each spouse’s vulnerabilities were defined as a latent variable with three indicators: abuse history, environment in the family-of-origin, and negative emotionality. *Abuse history* was conceptualized as spouses’ experience with maltreatment, which included sexual abuse and physical abuse. Individuals were identified as having a history of physical abuse with three self-report items assessing victimization in different contexts. E.g., “Before you turned 18, were you ever hit, beaten up, burned, assaulted with a weapon, or had your life threatened by a member of your family?” Individuals were identified as having a history of sexual abuse from items assessing victimization before as well as after the ages of 18 (i.e., “did anyone—a stranger, friend, acquaintance, date, or relative—ever try or succeed in doing something sexual to you or make you do something sexual to them against your wishes?”). Type of abuse was summed into one global measure of abuse history with scores ranging from 0 to 3. *Family environment in childhood* was assessed with three items measuring the closeness and happiness of the family prior to age 14 (e.g., “My parents’ relationship would be a good example to follow for any married couple,” “The members of my family were always very close to each other”), with 0 = true and 1 = false, as well as a fourth item assessing whether participants’ parents were divorced/separated versus intact. Scores for the four items were summed to form the family environment score for each participant, with an alpha of 0.67 for husbands and 0.66 for wives. *Symptoms of depression*, an indicator of negative emotionality, were assessed by summing 9 items from the CESD (Radloff, 1977) measuring dysphoria, sadness, and depression over the past 30 days (e.g., “During the last 30 days, about how often did you feel so sad that nothing could cheer you up?”), with an alpha of 0.75 for husbands and 0.73 for wives.

#### Stress

Chronic stress was assessed by items identifying typical conditions in each of eight domains: close friendships, intimate relationship, extended family relationships, relationships with children, work, finances, health of self, and health of family members, which were summed to create an overall chronic stress variable (Hammen et al., 2012). Husbands’ and wives’ scores were then summed to form the couple level chronic stress score. The majority of couples were not administered the chronic stress measure at Time 1 (*n* = 330); the chronic stress measure was administered to Black and White couples at Time 1 but only to Hispanic couples starting at Time 2. For couples who did provide chronic stress data at Time 1 (*n* = 101), their reports of chronic stress at Time 1 and Time 2 were correlated (husbands’ *r* = 0.56, *p <* 0.001, wives’ *r* = 0.66, *p <* 0.001), suggesting that this measure of chronic stress is indeed capturing a relatively stable phenomenon, consistent with our conceptualization of stress as chronic. Given the reasonably stable nature of the perceived stress measured by this variable, for couples with missing data, Time 2 reports of chronic stress were imputed into the their Time 1 data.

#### Adaptive processes

Couple adaptive processes were measured by six observed indicators of communication during an interactional task at Time 1: husband and wife positivity, husband and wife effectiveness, and husband and wife negativity (reverse-coded). A composite *positivity* scale was created by averaging an individual’s scores on the group enjoyment, positive mood, warmth/support, physical affection, humor/laugh, endearment, and listener responsiveness codes in each task, and then averaging across the three tasks; ICC = 0.83 for husbands, 0.81 for wives. A composite *effectiveness* scale was created by averaging an individual’s scores on the assertiveness, communication, effective process, solution quality, and solution quantity in each task, and then averaging across the three tasks; ICC = 0.74 for husbands, 0.80 for wives. A composite *negativity* scale was created by averaging an individual’s scores on the hostility, disruptive process, contempt, denial, angry coercion, dominance, verbal attack, interrogation, and externalized negative codes in each task, and then averaging across the three tasks; ICC = 0.73 for husbands, 0.74 for wives. The negativity score was reverse-coded in order to be included in the latent variable along with positivity and effectiveness.

#### Relationship satisfaction

Relationship satisfaction, conceptualized as spouses’ global sentiment toward the relationship, was assessed by summing responses on an eight-item questionnaire. The measure was adapted from Rauer et al. (2008) and included items from the General Social Survey (Davis et al., 2006). It has been used in large surveys with low-income couples (e.g., Rauer et al., 2008) and has been shown to covary systematically with observed communication, thus lending support to its validity as an indicator of relationship functioning (Williamson et al., 2013). Five items asked how satisfied the respondent was with various areas of their relationship (e.g., time spent together) and were scored on a 5-point scale (1 = *Very dissatisfied*, 5 = *Very satisfied*). Three items asked to what degree the participant agreed with a statement about their relationship (e.g., “how much do you trust your partner?”) and were scored on a 4-point scale (1 = *Not at all*, 4 = *Completely*). Scores could range from 8 (very dissatisfied) to 37 (very satisfied). Coefficient α exceeded .70 for husbands and wives at all assessments.

#### Sociodemographic risk

Sociodemographic risk, our hypothesized moderator variable, was assessed using a 10-item index developed by Amato (2014). Couples were given 1 point for the presence of each of the following items: (a) either partner was under the age of 23, (b) husband had less than a high school education, (c) wife had less than a high school education, (d) husband was unemployed, (e) wife was unemployed, (f) couple’s income was below the poverty line, (g) husband was receiving public assistance, (h) wife was receiving public assistance, (i) husband reported no one to help in an emergency, and (j) wife reported no one to help in an emergency. Prior work shows that this index moderates the effects of skills training on observed communication 18 months later (Williamson et al., 2016).

#### Analytic plan

All three aims were tested using latent variable structural equation models (SEM), as SEM allows for the creation of latent variables using multiple measured variables as indicators (thereby accounting for the measurement error in each of the observed variables and yielding more precise regression coefficients). In addition, SEM accounts for the dependency between spouses and allows for all independent and dependent variables to be tested simultaneously (thereby estimating the effects of each variable over and above all others). We obtained maximum likelihood estimates of the model coefficients using MPlus Version 7.0.

We evaluated three structural equation models. In the first, three latent factors (husband vulnerabilities, wife vulnerabilities, and couple adaptive processes), and one observed variable (couple chronic stress), were used to predict the intercepts and slopes of relationship satisfaction, simultaneously for husbands and for wives. In the second model, we examined whether adaptive processes would mediate effects of vulnerabilities and stress on satisfaction. The third model was similar to the first but added the index of sociodemographic risk, as an observed variable, to determine whether associations between the three predictor variables (vulnerabilities, stress, and adaptive processes) and the two dependent variables (satisfaction intercepts and slopes) varied systematically as a function of risk.

## Results

### Descriptive statistics and preliminary analyses

Descriptive statistics for all study variables, and their estimated loadings on our three latent factors, are shown in Table 1, and correlations among our primary study variables are shown in Table 2. Correlations among the three latent variables in the model were generally small but in expected directions (*r* = |0.17–0.26|). Adaptive processes were unrelated to chronic stress, a couple-level measured variable (*r* = −0.04, *ns*), whereas enduring vulnerabilities did covary with chronic stress, for husbands (*r* = 0.53, *p* < 0.001) and for wives (*r* = 0.54, *p* < 0.001). When considering VSA model components in relation to the index of sociodemographic risk, Table 2 shows that people with fewer social and economic resources enter marriage with more enduring vulnerabilities (*r* = 0.36 for husbands, *p* < 0.001; *r* = 0.16 for wives, *p* < 0.05) and poorer adaptive processes (*r* = −0.39, *p* < 0.001) and more chronic stress (*r* = 0.12, *p* < 0.01). It should be noted that variability in sociodemographic risk in our sample (*SD* = 2.12) was significantly higher [*F(387, 7923)* = 1.37, *p* < 0.0001] than the variability originally reported in Amato’s (2014) sample (*SD* = 1.81).

**TABLE 1:** Descriptive statistics and factor loading estimates for study variables.

Construct	Variable	M	SD	Factor loading estimate
Husband vulnerability	Abuse history	0.46	0.77	0.24**
	Family environment	1.01	1.12	0.19**
	Symptoms of depression	4.00	3.32	0.59***
Wife vulnerability	Abuse history	0.70	1.00	0.43***
	Family environment	1.12	1.13	0.46***
	Symptoms of depression	3.70	3.03	0.52***
Adaptive processes	Husband positivity	2.38	0.78	0.51***
	Wife positivity	2.35	0.76	0.54***
	Husband effectiveness	4.18	0.89	0.37***
	Wife effectiveness	4.29	0.87	0.41***
	Husband negativity	3.09	0.59	0.38***
	Wife negativity	3.06	0.58	0.31***
Relationship satisfaction	Husband T1 satisfaction	33.90	3.05	
	Wife T1 satisfaction	33.15	3.39	
	Husband T2 satisfaction	33.43	3.71	
	Wife T2 satisfaction	32.83	3.69	
	Husband T3 satisfaction	33.44	3.50	
	Wife T3 satisfaction	32.38	4.08	
	Husband T4 satisfaction	33.02	4.05	
	Wife T4 satisfaction	32.30	4.15	
Couple chronic stress	Couple chronic stress	8.51	4.10	
Sociodemographic risk	Sociodemographic risk	2.38	2.12	

For Factor Loading Estimates, *p < 0.05, **p < 0.01, ***p < 0.001. The above loadings were derived from confirmatory factor analysis. To evaluate whether any these variables cross-loaded on other factors, we also conducted an exploratory factor analysis. Modification indices in this analysis did not indicate that any variables loaded on a second factor. Moreover, and as detailed in the text, CFI, TLI, and SRMR suggest strong fit for the confirmatory model, suggesting that cross-loadings are not undermining this model.

**TABLE 2:** Correlation matrix for latent variables and observed variables in final model.

	Variable	1	2	3	4	5	6	7	8	9
1	Husband vulnerabilities	1								
2	Wife vulnerabilities	0.26*	1							
3	Adaptive processes	−0.24*	–0.17	1						
4	Stress	0.53***	0.54***	–0.04	1					
5	Husband satisfaction intercept	−0.55***	−0.27***	0.41***	−0.43***	1				
6	Wife satisfaction intercept	−0.29***	−0.51***	0.53***	−0.37***	0.47***	1			
7	Husband satisfaction slope	0.25	–0.03	–0.11	–0.05	0.27*	0.37*	1		
8	Wife satisfaction slope	–0.03	–0.20	–0.06	–0.10	0.17	0.45*	0.55*	1	
9	Sociodemographic risk	0.36***	0.16*	−0.39***	0.12*	−0.13*	−0.15**	0.01	−0.26*	1

*p < 0.05, **p < 0.01, ***p < 0.001.

Two important points can be drawn from these analyses. First, evidence that chronic stress is unrelated to couple communication (*r* = −0.03, *ns*) is at odds with VSA model predictions. However, sociodemograpic risk and couple communication do covary reliably (*r* = −0.38, *p* < 0.001), indicating that there may be contextual constraints on adaptive processes (or that poorly communicating couples select themselves into difficult circumstances). Second, evidence that chronic stress and sociodemographic risk are weakly associated (*r* = 0.12, *p* < 0.010) indicates that stress as assessed here is distributed evenly across the sociodemographic spectrum; that is, there are couples with high levels of chronic stress who also possess high levels of social and economic capital, just as there are couples with low levels of chronic stress who confront high levels of social and economic vulnerability. Both findings corroborate the separability of chronic stress and sociodemographic risk, supporting our plan to examine sociodemographic risk as a moderator of paths linking VSA model components to satisfaction trajectories (i.e., Aim 3).

Turning to correlations involving our satisfaction outcomes, Table 2 shows that spouses with more enduring vulnerabilities had lower satisfaction intercepts (*r* = −0.55, *p* < 0.001 for husbands; *r* = −0.51, *p* < 0.001 for wives), as did couples who experienced more chronic stress (*r* = −0.43, *p* < 0.001 for husbands*; r* = −0.37, *p* < 0.001 for wives). Couples displaying higher-quality adaptive processes also had higher satisfaction intercepts (*r* = 0.41, *p* < 0.001 for husbands; *r* = 0.53, *p* < 0.001 for wives). VSA predictor variables were unrelated to satisfaction slopes, for husbands and for wives. Overall, these bivariate associations corroborate VSA model predictions and replicate established effects in research on couples. At the same time these results leave open questions about whether these three factors independently predict outcomes (Aim 1) and whether adaptive processes function as a mediator (Aim 2).

For the baseline model (with and without mediation), with no moderation, the model fit the data well, exceeding the minimum value of.95 for the comparative fit index (CFI) and achieving good fit (<0.05) indexed by the root mean square error of approximation and standardized root mean square residual (< 0.08), in accordance with suggestions made by Hu and Bentler (1999) for a good model fit (CFI = 0.96, TLI = 0.95, RMSEA = 0.04, SRMR = 0.07). The longitudinal moderation involved in the modeling required an optimization algorithm with numerical integration, and this type of model does not yield model fit indices.

### Aim 1: Independent effects of Vulnerability-Stress-Adaptation Model components on relationship satisfaction

**FIGURE 1:**
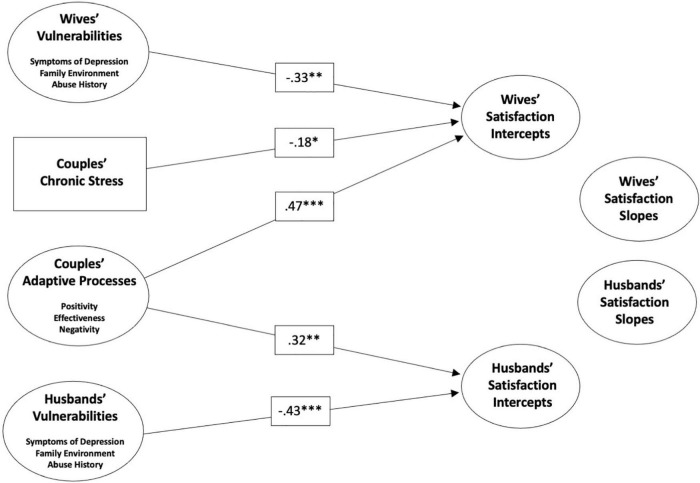
Structural equation model relating vulnerabilities, chronic stress, and couples’ adaptive processes to wives’ and husbands’ satisfaction intercepts and slopes. Only significant paths are shown. *N* = 388 couples. *p < 0.05, ^**^p < 0.01, ^***^p < 0.001.

As shown in Figure 1, wives’ higher levels of enduring vulnerabilities (β = −0.33, *p* < 0.01), higher levels of chronic stress (β = −0.18, *p* < 0.05), and poorer adaptive processes (β = 0.47, *p* < 0.001) all independently predicted lower satisfaction intercepts for wives. For husbands’ satisfaction intercepts, similar effects were evident for enduring vulnerabilities (β = −0.43, *p* < 0.01), and for adaptive processes (β = 0.32, *p* < 0.01), but not for chronic stress (β = −0.16, *ns*). As was the case with our bivariate correlations, changes in satisfaction were unrelated to vulnerabilities, stress, and adaptive processes, for wives and for husbands.^1^

In sum, while VSA model components did not predict the extent to which satisfaction declined for any given couple, they did reliably differentiate among couples at different levels of relationship satisfaction, albeit with unexpectedly non-significant effects of chronic stress on husbands’ satisfaction levels.

### Aim 2: Mediated effects of Vulnerability-Stress-Adaptation Model components on relationship satisfaction

Consistent with the original formulation of the original VSA model, we next computed a structural equation model in which the effects of stress and enduring vulnerabilities on satisfaction slopes and intercepts were mediated by adaptive processes. The model tested indirect effects of stress and vulnerabilities mediated by adaptive processes on husbands’ and wives’ intercepts and slopes of satisfaction simultaneously using 5000 bootstrap simulations. We elected bootstrapping methods for testing indirect effects because of their superior statistical power compared to traditional techniques (Zhao et al., 2010).

All indirect effects in this mediational model were nonsignificant. For wives, the indirect effect of couples’ stress mediated by adaptive processes on both the intercepts and slopes were non-significant (β = 0.13, *ns* and β = −0.03, *ns*, respectively). Moreover, the effects of wives’ vulernabilities on wives’ satisfaction intercepts and slopes were not mediated by adaptive processes (β = −0.10, *ns* and β = 0.02, *ns*, respectively). Similarly for husbands, the effects of couple-level stress and husbands’ vulnerabilities on satisfaction intercepts and slopes were not significantly mediated by adaptive processes (β = 0.09, *ns* for stress on husband intercept; β = −0.11, *ns* for husband vulnerability on husband intercept; β = −0.01, *ns* for stress on husband slope; β = 0.02, *ns* for husband vulnerability on husband slope).

### Aim 3: Moderating effects of sociodemographic risk on Vulnerability-Stress-Adaptation Model paths

Earlier we documented the low degree of overlap between sociodemographic risk and chronic stress (*r* = 0.12), underscoring the distinguishability of these concepts. To test Aim 3 we estimated a second structural equation model using this risk index as a moderator of all paths linking VSA model components with relationship satisfaction intercepts and slopes. In this model, risk was unrelated to satisfaction intercepts (βs = −0.17 and 0.12, for husbands and wives, respectively) and satisfaction slopes (βs = 0.02 and –0.44, for husbands and wives; all *ns*).

**FIGURE 2:**
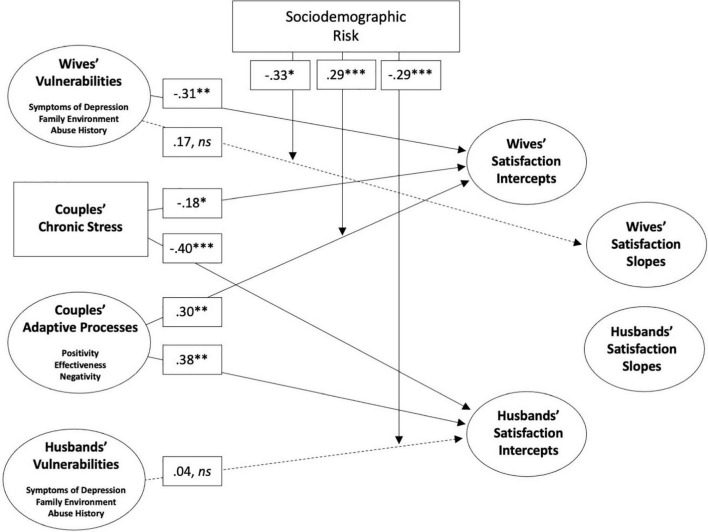
Structural equation model showing moderating effects of sociodemographic risk on associations relating vulnerability, stress, and adaptive processes to husbands’ and wives’ satisfaction intercepts and slopes. Significant paths are shown. Dashed paths indicate non-significant main effects that were moderated by sociodemographic risk. *N* = 388 couples. *p < 0.05, ^**^p < 0.01, ^***^p < 0.001.

Results of the second structural equation, shown in Figure 2, are very similar to those obtained with our base model (Figure 1). Specifically, four significant main effects remain significant even with these interaction terms in the model, demonstrating again that wives’ satisfaction intercepts are reliably lower when they experience more vulnerabilities (β = −0.31, *p* < 0.01) and more chronic stress (β = −0.18, *p* < 0.05), and that husbands’ and wives’ satisfaction intercepts are reliably lower to the extent they display poorer adaptive processes (β = 0.38, *p* < 0.01, and β = 0.30, *p* < 0.05, respectively). One new main effect emerges, indicating that husbands’ intercepts are lower to the extent they experience more chronic stress (β = −0.40, *p* < 0.001).

**FIGURE 3:**
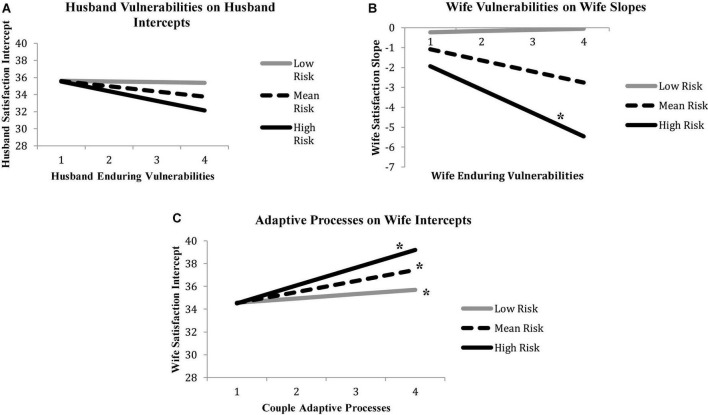
Sociodemographic risk interacts with **(A)** husbands’ vulnerabilities to predict husband satisfaction intercepts, **(B)** wives’ vulnerabilities to predict wives’ satisfaction slopes, and **(C)** adaptive processes to predict wives’ satisfaction intercepts. Asterisks denote statistically significant estimates of simple slopes.

In three instances, sociodemographic risk moderated associations with satisfaction outcomes. These three effects are shown in Figure 2, and analyses of simple slopes are provided in Figure 3. First, among husbands, sociodemographic risk strengthened the inverse association between husbands’ vulnerabilities and their own relationship satisfaction intercept (β = −0.29, *p* < 0.001). Simple slopes for values at the mean and 1 *SD* above and below the mean were calculated and tested against zero for significance. When sociodemographic risk was 1 *SD* below the sample mean, husbands’ vulnerabilities were unrelated to their satisfaction intercepts (β = −.13, *ns*). However, this association grew stronger as sociodemographic risk increased from the sample mean (β = −1.82, *p* < 0.01) to 1 *SD* above the mean (β = −3.52, *p* < 0.01).

Second, among wives, sociodemographic risk moderated the association between wives’ vulnerabilities and their own satisfaction slopes (β = −0.33, *p* < 0.05). When sociodemographic risk was 1 *SD* below the sample mean and at the sample mean, wives vulnerabilities were unrelated to their satisfaction slopes (β = 0.16 and β = −0.21, respectively, both *ns*). However, at the highest level of sociodemographic risk, wives’ enduring vulnerabilities reliably foreshadowed steeper drops in satisfaction (β = −0.57, *p* < 0.05).

Finally, sociodemographic risk moderated the association between observed adaptive processes and wives’ satisfaction intercepts (β = 0.29, *p* < 0.001). Estimation of simple slopes for this interaction revealed that better adaptive processes covaried with higher intercepts for wives across all levels of sociodemographic risk, and that this association became stronger as sociodemographic risk increased: β = 2.17 for low-risk couples; β = 4.09 for average-risk couples; β = 6.00 for high-risk couples (all *p*-values < 0.01).

In sum, out of 8 possible interactions involving *intercept* effects (4 independent variables x husbands’ and wives’ intercepts), only 2, or 25%, were statistically significant. With *slope* effects, 1 of 8 possible interactions, or 12.5%, were statistically significant, though few slope effects in general were observed. Thus most associations within the VSA model generalize across levels of sociodemographic risk and, in those three instances when moderation did occur, effects were consistently stronger among couples with fewer social and economic resources.

## Discussion

Why do some marriages falter where others thrive? Integrating across several established literatures in relationship science, the Vulnerability-Stress-Adaption (VSA) Model identifies three distinguishable predictor domains and argues that couples are more likely to struggle when partners enter marriage with more turbulent and unstable family backgrounds as well as potentially distress-generating emotional tendencies (e.g., trait-like propensities to experience negative emotion, symptoms of depression), when couples inhabit ecological niches characterized by higher levels of stress, and when partners communicate with more negativity and less sensitivity. Using a large and diverse sample, we demonstrate—consistent with VSA model predictions—that enduring vulnerabilities, chronic stress, and poor adaptive processes all independently predict lower relationship satisfaction (Aim 1). Among the three domains, none emerged as obviously stronger in magnitude, though the case could be made that results obtained with adaptive processes are particularly noteworthy: assessed with a brief and perhaps somewhat artificial interaction task, and evaluated by outside coders, objectively-rated adaptive processes predicted satisfaction at least as well as self-report indices of stress and enduring vulnerabilities. More generally, however, these findings (a) corroborate the view that the VSA model can be meaningfully applied to data collected from diverse couples and (b) lend support to the view that relationship satisfaction is multiply-determined, thus helping to rule out the idea that any single domain overrides the influence of other predictors.

The VSA model goes further in assigning adaptive processes a central and proximal role in determining partners’ evaluations of their relationship, predicting that associations relating enduring vulnerabilities and stress to satisfaction outcomes will be mediated by couple communication. That is, the manner in which partners communicate is expected to reflect stable elements of their personalities and early experiences, and the stress they are under, such that the effects of enduring vulnerabilities and stress on relationship outcomes will be lessened when communication is also included in the model (Aim 2). Despite relatively robust main effects for adaptive processes, we found no evidence that the behavioral data performed as a mediator, casting doubt on a central assumption of the VSA model. As we elaboraborate below, these findings raise new questions about how enduring vulnerabilities and stress may come to be associated more directly with judgments of relationship satisfaction.

Finally, Aim 3 addressed whether the magnitude of VSA model paths was invariant across couples’ levels of social isolation and economic deprivation, or whether such paths might grow stronger for those couples with greater sociodemographic vulnerability. After establishing that chronic stress was well-distributed at all levels of sociodemographic risk, we demonstrated that husbands’ and wives’ enduring vulnerabilities, and their adaptive processes, were indeed associated more closely with satisfaction at higher than at lower levels of sociodemographic risk. Thus, otherwise identical personal backgrounds and communication behaviors appear to be more consequential for disadvantaged couples, underscoring the need for models that (a) explicitly acknowledge couples’ socioeconomic constraints and (b) more specifically recognize the possibility that the adaptive processes needed to sustain a satisfying relationship may differ as a function of these constraints (also see Ross et al., 2019). Additional support for understanding couples within specific environments comes from our correlational finding (Table 2) that the quality of observed communication was poorer for couples higher in sociodemographic risk. While it bears noting that moderating effects were nonsignificant in most of the tested interactions, the point remains that the magnitude of at least some VSA model paths do vary as a function of the social and economic disadvantages that couples face.

### Limitations

Several factors temper enthusiasm for these findings. First, with the exception of the moderated association beween wives’ enduring vulnerabities and their slopes, all statistically significant main effects and interactions were obtained only for satisfaction intercepts (i.e., satisfaction at baseline). Although a longer follow-up interval might have yielded more effects on slopes/trajectories, predicting changes in satisfaction has proven to be difficult (e.g., Lavner et al., 2014), perhaps because satisfaction itself is a relatively stable variable or because we relied on time-invariant independent variables collected at baseline (cf. McNulty et al., 2021). Second, strong causal claims are unwarranted. Inferences of causation are perhaps strongest for key enduring vulnerabities (e.g., experiences in the family of origin), which do precede entry into the relationships studied here, and reliably foreshadow lower satisfaction intercepts for husbands and wives. These are correlational effects, however, and third-variable effects cannot be fully eliminated. Inferences of causation must be even more tentative for the effects of chronic stress and communication, which unfold concurrently with satisfaction and are likely to be reciprocally related to satisfaction. More frequent assessments, and experiments undertaken to change communication or chronic stress (as might be done with studies of universal basic income, for example) will be needed to disentangle likely causes and effects.

Third, we have not provided a definitive nor broad test of the VSA model. Our analysis operationalized vulnerabilities, stress, and adaptive processes in specific ways, and future research that incorporates more variables into in three VSA domains is needed. Similarly, we have not provided a definitive test of the mediational role played by adaptive processes. This is intended as a broad concept in the VSA model, yet in the field generally, and in our analysis specifically, adaptive processes were operationally defined using a series of relatively brief interaction tasks. The various interdependencies that characterize the full range of couples’ interpersonal experiences is likely to extend well beyond such structured tasks, and alternative paradigms are needed to provide additional insight into adaptive processes and their relation to enduring vulnerabilities and chronic stress (e.g., diary studies or naturalistic observation). Such data are needed before we can be confident that enduring vulnerabilities and stress exert effects on satisfaction that are unmediated by adaptive processes, broadly defined. Finally, while a diverse sample of different-sex couples provided data for this study, we leave vast domains of diversity remain unexplored, and conclusions cannot be readily generalized to, e.g., older couples, unmarried and remarried couples, or same-sex couples. VSA concepts likely extend to many forms of committed adult partnerships, but couples vary widely in the environments to which they are exposed, and thus generalizability cannot be assumed.

### Implications for theory, research, and intervention

Notwithstanding these concerns, the present findings may have implications for how we explain, study, and prevent relationship distress. For example, by taking seriously the finding that enduring vulnerabilities and stress are not fully mediated by adaptive processes, we might consider the possibility that personal backgrounds and stress *directly* alter the ways in which people appraise their relationship. Coming from a background of divorce, for example, is known to lower the threshold of marital distress required to instigate thoughts of divorce in one’s own relationship (Amato and DeBoer, 2001), and exposure to higher levels of stress causes partners to evaluate their relationship problems more critically—even as those problems remain unchanged (Neff and Karney, 2004). Thus, rather than necessarily affecting overt communication processes, there may be mechanisms by which enduring vulnerabilities and stress circumvent interpersonal processes to directly influence how judgments of satisfaction are organized or triggered. Spouses’ positive and negative behaviors in standard interaction tasks are surprisingly stable over the early years of marriage, even as satisfaction drops (Williamson and Lavner, 2020; Williamson, 2021), suggesting that deteriorating construals of interactional processes, moreso than the processes themselves, contribute to declining satisfaction. Future studies are needed to examine whether enduring vulnerabilities and chronic stress might govern these construals, perhaps using new methods to assess satisfaction that minimize self-report bias (e.g., implicit measures of automatic attitudes; see McNulty et al., 2013). At the same time, we must emphasize that adaptive processes have been found to mediate effects of enduring vulnerabilities and stress on satisfaction, in a large sample of predominantly White couples (McNulty et al., 2021); additional analyses are needed to clarify whether the discrepant findings are due to sample characteristics, observational tasks and coding, or some other factor.

Although we did not study a treatment-seeking sample of couples, our findings may have relevance for interventions aimed at strengthening relationships—and, in particular, may help explain why prevention programs yield few lasting effects (for a review, see Bradbury and Bodenmann, 2020). Virtually all prevention programs focus on couple communication as the primary target of change, but the findings presented here indicate that adaptive processes do not predict *changes* in satisfaction, and that satisfaction is reliably associated as well with enduring vulnerabilities and chronic stress. Thus, even if lasting improvements in communication were possible, satisfaction may not stabilize because (a) poor communication may not be a true cause of satisfaction, (b) some couples will have entered marriage with risky personal backgrounds, and (c) once married, these same couples are most likely to experience high levels of chronic stress. Even if enduring vulnerabilities and chronic stress do not formally cause declines in satisfaction, these factors may constrain and perhaps even neutralize the effects of communication-based interventions. By recognizing the various factors that covary with relationship distress, however, other approaches become salient. For example, it may prove efficient to focus specifically on people with more enduring vulnerabilities, and particularly those living with high levels of sociodemographic risk, given the combined impact of these two factors on satisfaction (Figures 3A,B). Because improvements in communication quality could have disproportionately large benefits for high-risk couples (Figure 3C), our results support testing interventions for disadvantaged couples who enter committed partnerships with risky backgrounds, with emphasis on reducing the chronic stress that compromises their relationship functioning (Doss et al., 2016; Barton et al., 2018).

In sum, we demonstrate with a large and diverse sample that between-couple variation in marital satisfaction is a function of the trait-like tendencies and experiences that partners would likely bring to any relationship, the quality of communication they display, and the chronic stress they encounter as a couple. Adaptive processes performed well but did not mediate effects of enduring vulnerabilities or stress on satisfaction. These findings corroborate key predictions of the VSA model with data from couples living with low incomes and, by challenging key assumptions of this model, invite new research on the determinants of relationship satisfaction.

## Data availability statement

The raw data supporting the conclusions of this article will be made available by the authors, without undue reservation.

## Ethics statement

Sampling and data collection procedures were reviewed and approved by the RAND Corporation. The patients/participants provided their written informed consent to participate in this study.

## Author contributions

JR conducted data analyses and wrote the first draft of the manuscript. TN conducted additional analyses and edited the manuscript. BK was PI on the grant that funded data collection, conceived the model tested in the manuscript, co-supervised observational coding, and edited the manuscript. TB was Co-I on the grant supporting the work, conceived the model tested in the manuscript, supervised data analyses, co-supervised observational coding, and revised and edited the manuscript. All authors contributed to the article and approved the submitted version.

## Footnotes

1 In a separate analysis, a similar model was run that also included partner effects of enduring vulnerabilities (i.e., effect of spouse A’s vulnerabilities on spouse B’s outcomes, and vice versa). All four partner effects in this model were nonsignificant, when predicting wife intercepts (β = −0.17, *p* = 0.27) and slopes (β = 0.20, *p* = 0.49) and when predicting husband intercepts (β = −0.06, *p* = 0.59) and slopes (β = −0.30, *p* = 0.11. We therefore focus our analysis on actor effects.

## References

[B1] Amato P. R. (1996). Explaining the intergenerational transmission of divorce. *J. Marriage Family* 58 628–640.

[B2] Amato P. R. (2014). Does social and economic disadvantage moderate the effects of relation education on unwed couples? an analysis of data from the 15-month building strong families evaluation. *Family Relations* 63 343–355. 10.1111/fare.12069

[B3] Amato P. R. DeBoer D. D. (2001). The transmission of marital instability across generations: relationship skills or commitment to marriage? *J. Marriage Family* 63 1038–1051. 10.1111/j.1741-3737.2001.01038.x

[B4] Barton A. W. Beach S. R. H. Wells A. C. Ingels J. B. Corso P. S. Sperr M. C. (2018). The protecting strong african american families program: a randomized controlled trial with rural african American couples. *Prev. Sci.* 19 904–913. 10.1007/s11121-018-0895-4 29629507 PMC6177321

[B5] Bodenmann G. (2005). “Dyadic coping and its significance for marital functioning,” in *Couples Coping with Stress*, eds Revenson T. Kayser K. Bodenmann G. (Washington, DC: APA), 33–50. 10.1037/11031-002

[B6] Bodenmann G. Meuwly N. Germann J. Nussbeck F. W. Heinrichs M. Bradbury T. N. (2015). Effects of stress on the social support provided by men and women in intimate relationships. *Psychol. Sci.* 26 1584–1594. 10.1177/0956797615594616 26341561

[B7] Bowlby J. (1979). *The Making and Breaking of Affectional Bonds.* London: Tavistock.

[B8] Bradbury T. N. Bodenmann G. (2020). Interventions for couples. *Annual Rev. Clin. Psychol.* 16 99–123. 10.1146/annurev-clinpsy-071519-020546 32031866

[B9] Bramlett M. D. Mosher W. D. (2002). Cohabitation, marriage, divorce, and remarriage in the United States. *Vital Health Stat.* 23 1–93. 12183886

[B10] Conger R. D. Elder G. H. Jr. Lorenz F. O. Conger K. J. Simons R. L. (1990). Linking economic hardship to marital quality and instability. *J. Marriage Family* 52 643–656. 10.1192/bjp.174.2.112 10211164

[B11] Copen C. E. Daniels K. Vespa J. Mosher W. D. (2012). First marriages in the United States: data from the 2006–2010 national survey of family growth. *Natl. Health Statistics Rep.* 49 1–22. 22803221

[B12] Cutrona C. E. (1996). *Social Support in Marriage.* Thousand Oaks, CA: Sage.

[B13] Davis J. A. Smith T. W. Marsden P. V. (2006). *General Social Surveys, 1972-2006: Cumulative Codebook*. Chicago, IL: National Opinion Research Center.

[B14] Doss B. D. Cicila L. N. Georgia E. J. Roddy M. K. Nowlan K. M. Benson L. A. (2016). A randomized controlled trial of the web-based our relationship program: effects on relationship and individual functioning. *J. Consult. Clin. Psychol.* 84 285–296. 10.1037/ccp0000063 26999504 PMC4804631

[B15] Doss B. D. Rhoades G. K. Stanley S. M. Markman H. J. (2009). The effect of the transition to parenthood on relationship quality: an 8-year prospective study. *J. Pers. Soc. Psychol.* 96 601–619. 10.1037/a0013969 19254107 PMC2702669

[B16] Dronkers J. Härkönen J. (2008). The intergenerational transmission of divorce in cross-national perspective: results from the fertility and family surveys. *Population Stud.* 62 273–288. 10.1080/00324720802320475 18937142

[B17] Fisher T. D. McNulty J. K. (2008). Neuroticism and marital satisfaction: the mediating role played by the sexual relationship. *J. Fam. Psychol.* 22 112–122. 10.1037/0893-3200.22.1.112 18266538

[B18] Gottman J. M. Levenson R. W. (1986). Assessing the role of emotion in marriage. *Behav. Assess.* 8 31–48.

[B19] Hammen C. Hazel N. A. Brennan P. A. Najman J. (2012). Intergenerational transmission and continuity of stress and depression: depressed women and their offspring in 20 years of follow-up. *Psychol. Med.* 42 931–942. 10.1017/S0033291711001978 22018414 PMC3384497

[B20] Heyman R. E. Slep A. M. S. Lorber M. F. Mitnick D. M. Xu S. (2019). A randomized, controlled trial of the impact of the couple CARE for parents of newborns program on the prevention of intimate partner violence and relationship problems. *Prev. Sci.* 20 620–631. 10.1007/s11121-018-0961-y 30535623 PMC7193942

[B21] Hu L. T. Bentler P. M. (1999). Cutoff criteria for fit indexes in covariance structure analysis: conventional criteria versus new alternatives. *Struct. Equat. Model.* 6 1–55. 10.1080/10705519909540118

[B22] Jacobson N. S. Margolin G. (1979). *Marital Therapy: Strategies Based on Social Learning and Behavior Exchange Principles.* New York, NY: Brunner/Mazel.

[B23] Karney B. R. (2021). Socioeconomic status and intimate relationships. *Annu. Rev. Psychol.* 72 391–414. 10.1146/annurev-psych-051920-013658 32886585 PMC8179854

[B24] Karney B. R. Bradbury T. N. (1995). The longitudinal course of marital quality and stability: a review of theory, methods, and research. *Psychol. Bull.* 118 3–34. 10.1037/0033-2909.118.1.3 7644604

[B25] Kelly E. L. Conley J. J. (1987). Personality and compatibility: a prospective analysis of marital stability and marital satisfaction. *J. Pers. Soc. Psychol.* 52 27–40. 10.1037//0022-3514.52.1.27 3820076

[B26] Lavner J. A. Bradbury T. N. (2010). Patterns of change in marital satisfaction over the newlywed years. *J. Marriage Family* 72 1171–1187. 10.1111/j.1741-3737.2010.00757.x 21116452 PMC2992446

[B27] Lavner J. A. Karney B. R. Bradbury T. N. (2014). Relationship problems over the early years of marriage: stability or change? *J. Family Psychol.* 28 979–985. 10.1037/a0037752 25150369 PMC8310661

[B28] Lavner J. A. Williamson H. C. Karney B. R. Bradbury T. N. (2020). Premarital parenthood and newlyweds’ marital trajectories. *J. Fam. Psychol.* 34 279–290. 10.1037/fam0000596 31613117 PMC7102923

[B29] Liang B. Williams L. M. Siegel J. A. (2006). Relational outcomes of childhood sexual trauma in female survivors: a longitudinal study. *J. Interpers. Violence* 21 42–57. 10.1177/0886260505281603 16399923

[B30] Malouff J. M. Thorsteinsson E. B. Schutte N. S. Bhullar N. Rooke S. E. (2010). The five-factor model of personality and relationship satisfaction of intimate partners. *J. Res. Personal.* 44 124–127. 10.1080/01926187.2012.748549

[B31] McNulty J. K. Meltzer A. L. Neff L. A. Karney B. R. (2021). How both partners’ individual differences, stress, and behavior predict change in relationship satisfaction: extending the VSA model. *Proc. Natl. Acad. Sci. U S A.* 118:e2101402118. 10.1073/pnas.2101402118 34183417 PMC8271655

[B32] McNulty J. K. Olson M. A. Meltzer A. L. Shaffer M. J. (2013). Though they may be unaware, newlyweds implicitly know whether their marriage will be satisfying. *Science* 342 1119–1120. 10.1126/science.1243140 24288337

[B33] Melby J. Conger R. Book R. Rueter M. Lucy L. Repinski D. (1998). *The Iowa Family Interaction Rating Scales*, 5th Edn. Ames, IA: Iowa State University.

[B34] Neff L. A. Karney B. R. (2004). How does context affect intimate relationships? linking external stress and cognitive processes within marriage. *Personal. Soc. Psychol. Bull.* 30 134–148. 10.1177/0146167203255984 15030629

[B35] Pasch L. A. Bradbury T. N. (1998). Social support, conflict, and the development of marital dysfunction. *J. Consult. Clin. Psychol.* 66 219–230. 10.1037//0022-006X.66.2.219 9583325

[B36] Pietromonaco P. R. Overall N. C. (2022). Implications of social isolation, separation, and loss during the COVID-19 pandemic for couples’ relationships. *Curr. Opin. Psychol.* 43 189–194. 10.1016/j.copsyc.2021.07.014 34416682 PMC8881098

[B37] Radloff L. S. (1977). The CES-D scale: a self-report depression scale for research in the general population. *Appl. Psychol. Measurement* 1 385–401. 10.1177/014662167700100306 26918431

[B38] Randall A. K. Bodenmann G. (2009). The role of stress on close relationships and marital satisfaction. *Clin. Psychol. Rev.* 29 105–115. 10.1016/j.cpr.2008.10.004 19167139

[B39] Rauer A. J. Karney B. R. Garvan C. W. Hou W. (2008). Relationship risks in context: a cumulative risk approach to understanding relationship satisfaction. *J. Marriage Fam.* 70, 1122–1135. 10.1111/j.1741-3737.2008.00554.x 19587840 PMC2706520

[B40] Righetti F. Faure R. Zoppolat G. Meltzer A. McNulty J. K. (2022). Factors that contribute to the maintenance or decline of relationship satisfaction. *Nat. Rev. Psychol.* 1 161–173. 10.1038/s44159-022-00026-2

[B41] Ross J. M. Karney B. R. Nguyen T. P. Bradbury T. N. (2019). Communication that is maladaptive for middle-class couples is adaptive for socioeconomically disadvantaged couples. *J. Pers. Soc. Psychol.* 116 582–597. 10.1037/pspi0000158 30321045 PMC10626985

[B42] Rusbult C. E. Verette J. Whitney G. A. Slovik L. F. Lipkus I. (1991). Accommodation processes in close relationships. *J. Pers. Soc. Psychol.* 60 53–78.

[B43] Sanders M. R. Halford W. K. Behrens B. C. (1999). Parental divorce and premarital couple communication. *J. Fam. Psychol.* 13 60–74. 10.1037/0893-3200.13.1.60

[B44] Story L. B. Karney B. R. Lawrence E. Bradbury T. N. (2004). Interpersonal mediators in the intergenerational transmission of marital dysfunction. *J. Fam. Psychol.* 18 519–529. 10.1037/0893-3200.18.3.519 15382977

[B45] United States Census Bureau (2002). *Summary Population and Housing Characteristics.* Washington, DC: U.S. Government Printing Office.

[B46] Weiss B. Lavner J. A. Miller J. D. (2018). Self-and partner-reported psychopathic traits’ relations with couples’ communication, marital satisfaction trajectories, and divorce in a longitudinal sample. *Personal. Disorders* 9 239–249. 10.1037/per0000233 27991812 PMC5476523

[B47] Williamson H. C. (2021). The development of communication behavior over the newlywed years. *J. Fam. Psychol.* 35 11–21. 10.1037/fam0000780 32658517 PMC7856059

[B48] Williamson H. C. Altman N. Hsueh J. Bradbury T. N. (2016). Effects of relationship education on couple communication and satisfaction: a randomized controlled trial with low-income couples. *J. Consult. Clin. Psychol.* 84 156–166. 10.1037/ccp0000056 26501497

[B49] Williamson H. C. Bornstein J. X. Cantu V. Ciftci O. Farnish K. A. Schouweiler M. T. (2021). How diverse are the samples used to study intimate relationships? a systematic review. *J. Soc. Personal Relationships* 39 1087–1109. 10.1177/02654075211053849 35655791 PMC9159543

[B50] Williamson H. C. Karney B. R. Bradbury T. N. (2013). Financial strain and stressful events predict newlyweds’ negative communication independent of relationship satisfaction. *J. Fam. Psychol.* 27 65–75. 10.1037/a0031104 23421833 PMC3667200

[B51] Williamson H. C. Lavner J. A. (2020). Trajectories of marital satisfaction in diverse newlywed couples. *Soc. Psychol. Personal. Sci.* 11 597–604. 10.1177/1948550619865056 34055202 PMC8153381

[B52] Woodin E. M. (2011). A two-dimensional approach to relationship conflict: meta-analytic findings. *J. Fam. Psychol.* 25 325–335. 10.1037/a0023791 21553964

[B53] Zhao X. Lynch J. G. Jr. Chen Q. (2010). Reconsidering Baron and Kenny: myths and truths about mediation analysis. *J. Consumer Res.* 37 197–206.

